# Molecular In Vivo Imaging Using a Noninvasive Cardiac-Specific MLC-2v Promoter Driven Dual-Gene Recombinant Lentivirus Monitoring System

**DOI:** 10.1371/journal.pone.0133952

**Published:** 2015-07-31

**Authors:** Miao Zhang, Lihua Wang, Rui Guo, Sheng Liang, Xufeng Jiang, Min Zhang, Biao Li

**Affiliations:** 1 Department of Nuclear Medicine, Rui Jin Hospital, School of Medicine, Shanghai Jiao Tong University, Shanghai, China; 2 Department of Nuclear Medicine, Xin Hua Hospital, School of Medicine, Shanghai Jiao Tong University, Shanghai, China; Emory University, UNITED STATES

## Abstract

**Background:**

Our study aimed to demonstrate the feasibility of using the sodium/iodide symporter (NIS) to monitor vascular endothelial growth factor (VEGF_165_) expression in vivo.

**Methods:**

We constructed a recombinant lentivirus plasmid with the MLC-2v promoter driving the sodium/iodide symporter (NIS) reporter gene linked to the VEGF_165_ gene. Expression of NIS and VEGF gene were identified by Western blot. On days 2 and 54, ^99m^Tc-MIBI imaging was used to evaluate changes in myocardial ischemia. Noninvasive ^125^I micro-SPECT/CT imaging was used to assess the expression of NIS reporter gene dynamically over the next 2 months.

**Results:**

Western blot analysis showed that both NIS and VEGF_165_ were highly expressed in rat cardiomyoblast H9C2 cells transduced with Lenti-MLC-2v-NIS--VEGF_165_. ^125^I micro-SPECT/CT reporter imaging showed higher uptake in mouse myocardium transduced with Lenti-MLC-2v-VEGF_165_-IRES-NIS. NIS expression peaked on day 1 after transduction followed by a progressive decline to negligible levels by day 21. On day 1, mean ^125^I activity value in group 1 was higher than that in group 2 (*P*<0.05). The mean ^125^I activity value in group 3 was statically lower than that in group 1 and 2 (*P*<0.01). On day 60, ^125^I uptakes in test and positive control groups became very low, and no significant differences in the mean ^125^I activity values were detected between group 1 and group 2 (*P* = 0.531 > 0.05). In group 1 (test group), ^99m^Tc-MIBI SPECT/CT revealed improvements in perfusion and wall thickening in the apical anterior wall. Mean IOD values of NIS and CD_34_ were significantly higher in group 1 than group 3 (*P*<0.05). Our study proved mean I-125 uptake was significantly correlated with mean IOD value of NIS and CD34 (*P*<0.05).

**Conclusion:**

This study demonstrates the feasibility of using the NIS gene to monitor VEGF_165_ expression in a mouse myocardial ischemia model.

## Introduction

Ischemic heart disease (IHD) is the leading cause of death worldwide [1,2]. Patients with ischemic HF continue to experience unacceptably high rates of morbidity and mortality. In some patients, even optimal subsequent medical treatment, such as coronary artery bypass grafting (CABG) surgery, cannot prevent the development of heart failure, which has triggered an increasing interest in novel therapeutic strategies [3]. Gene therapy for cardiovascular disease has great potential for a variety of application, but the lack of reliable monitoring methods has limited its development. Non-invasive molecular imaging to monitor the survival and location of gene expression in real-time is crucial for the success of gene therapy [4, 5].

Bioluminescent and fluorescent reporters [6, 7], although sensitive, suffer from limited light penetration in whole animal studies and relatively poor spatial resolution [8]. Radionuclide imaging reporters [5] have good levels of sensitivity and depth penetration. One of the first noninvasive reporter gene imaging paradigms was based on HSV1-tk. Recent studies using the HSV1-tk reporter gene to monitor gene or stem cells in myocardial infarction model show encouraging results [9, 10]. Sodium/iodide symporter (NIS) is another promising imaging reporter gene [11]. NIS has many advantages as a reporter gene, primarily because of the wide availability of substrates already approved for clinical use (both for γ-camera and PET imaging studies), and the fact that radiochemistry laboratory facilities do not need to be nearby [5]. Previous reports demonstrated the feasibility of using NIS for myocardial gene expression imaging in rats [12–14]. However, few reports have investigated the combination of NIS with therapeutic genes in myocardial ischemia in vivo.

Based on our previous work [15, 16], in this study, we used a dual gene recombinant lentivirus, which linked the NIS reporter gene with the VEGF_165_ therapeutic gene in an animal model of myocardial ischemia to assess the feasibility of the NIS gene for monitoring VEGF_165_ expression in vivo. In order to increase specificity of gene expression in a cardiac system, we used the promoter of the myosin light chain (MLC-2v) as this contractile protein is abundant in cardiac muscles [17, 18]. Although the MLC-2v promoter is 3 kb long, critical elements that mediate cardiac-specific gene expression are located within the first 250 bp [18]. The lentivirus system was chosen because this provides prolonged stable expression levels with lower in vivo immunogenicity than other systems based on baculovirus and adenovirus.

We hypothesize that the VEGF therapeutic gene expression can be monitored in vivo by the co-expression of the NIS reporter gene from a cardiac-specific promoter. This system would provide the basis of an effective method for monitoring the expression of other therapeutic proteins, such as FGF, HGF and HIF-1, in experimental or even clinical studies of myocardial ischemic disease.

## Materials and Methods

### Ethics statements

The study was approved by the institutional review board and the experimental animal center of Rui Jin Hospital, School of Medicine at the Shanghai Jiao Tong University.

### Recombinant virus generation and amplification

Construction of recombinant plasmids: The full-length sequences of NIS and VEGF cDNA were removed from the pcDNA_3_ expression vector (conserved by our laboratory) by restriction digestion followed by agarose gel purification. These sequences were then subcloned into plasmid Lenti-EF1a-Oct4-IRES-EGFP (kindly provided by Prof. Liu Mofang, Shanghai Institute for Biological Sciences), to generate plasmids pLenti-EF1a-NIS-IRES-GFP, pLenti-EF1a-VEGF_165_-IRES-NIS and pLenti-EF1a-VEGF_165_-IRES-GFP. Plasmid Lenti-MLC-2v-VEGF_165_-IRES-NIS was derived from plasmid Lenti-EF1a-VEGF_165_-IRES-NIS, in which the EF1a promoter was substituted by the myosin light chain (MLC-2v) promoter (250 bp) according to reference [18].

Lentivirus amplification: All lentiviral vectors were purified and amplified according to the manufacturer’s instructions with some modifications [19]. Virus titers were between 1×10^7^ and 1×10^8^ plaque-forming units (pfu). Lenti-null and Lenti-EF1a-VEGF_165_-IRES-NIS served as the negative and positive control viruses, respectively.

### Cell lines and cell cultures

Rat cardiomyoblast cells H9C2 and mouse skeletal myoblasts cells C2C12 obtained from the Chinese Academy of Sciences were maintained in Dulbecco’s modified Eagle’s medium (DMEM) supplemented with 10% fetal bovine serum (FBS) 100 U/mL penicillin and 100 mg/mL streptomycin and incubated with 5% CO_2_ atmosphere at 37°C.

### Western blot analysis of dual-gene expression in cells

H9C2 and C2C12 cells were seeded in 10-cm plates at a density of 4×10^5^cells/well and cultured with serum-free DMEM for 24 h. At 70% confluence, Lenti-MLC-2v-VEGF_165_-IRES-NIS and Lenti-EF1a-VEGF_165_-IRES-NIS were added (MOI = 40).

Lysates of lentivirus-infected cells were prepared by standard methods. Western blot analysis was then performed by incubating the filter with mouse anti-NIS (1:500; Neomarker), anti-His-tag (1:500; Neomarker) and anti-GAPDH (1:10000; Abgent) antibody in Tris-buffered saline Tween-20 overnight at 4°C, followed by incubation with peroxidase-conjugated goat anti-mouse immunoglobulin G (1:2500; Neomarker) for 1 h at room temperature. Immunodetection was carried out using an ECL Western blot detection kit (Pierce).

### Animal studies

Adult male BALB/C mice (weight, 20–25 g) were supplied by the Research Center for Experimental Medicine of Ruijin Hospital School of Medicine, Shanghai Jiaotong University (animal license number SYXK (HU) 2011–0113). All animals were caged under standard light and temperature conditions with free access to food and water throughout the study. All experimental procedures were made to minimize suffering which were approved by the local committee for animal welfare and were conducted in accordance with the approval of the Ethics Committee and Animal Care Committee of the School of Medicine at Shanghai Jiao Tong University. Our study was performed according to requirement of the ARRIVE guidelines for reporting animal research (S1 File).

Induction of myocardial infarction mouse model: Each mouse was anesthetized with 5% isofluorane in an induction chamber and then with 1–2% isofluorane gas delivered through a customized face-mask during surgery. The heart was exposed and myocardial infarction (MI) was induced by the permanent ligation at the distal third of the left anterior descending coronary artery with a 6–0 polypropylene suture. Subsequently, one spot in left ventricle anterior wall around the peri-infarct region was injected with a total of 20 μl of virus suspension or 0.9% saline.

**Fig 1:**
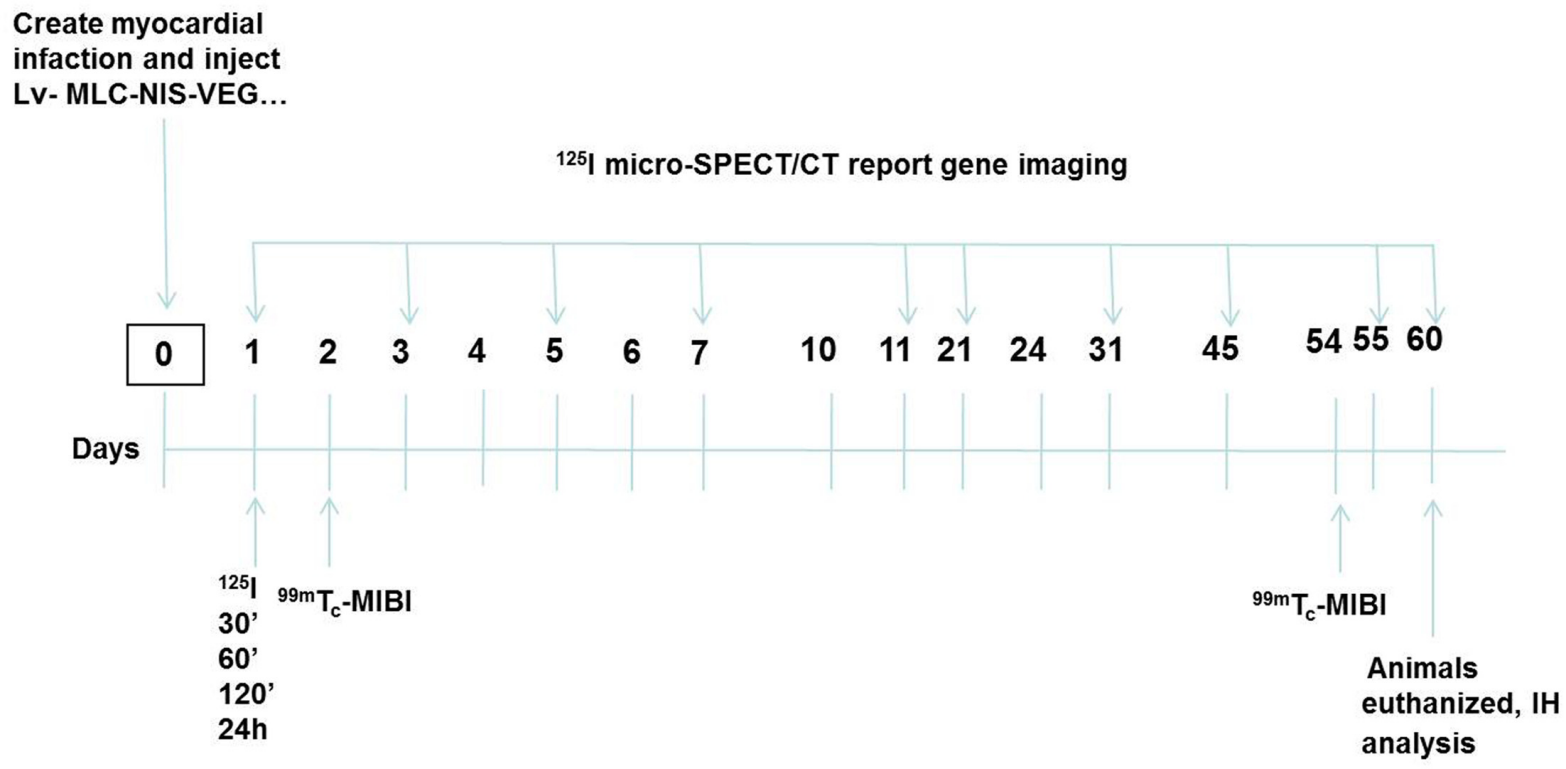
Outline of experimental design.

Forty-eight adult male BALB/C mice were used to establish the animal model. A total of twenty-six mice died in postoperative period because of acute myocardial infarction, arrhythmia or virus injection. Post-operative animal death for the twenty-six mice could not be predicted because mice were not showing signs of distress or discomfort, and therefore they could not be euthanized. The mortality in our study was anticipated and our Ethics and Animal Care Committee of the school of Medicine at Shanghai Jiao Tong University specifically reviewed and approved the mortality aspects of the study. To ensure equal randomization, the other twenty-two survival animals underwent ^99m^Tc-MIBI imaging at the baseline (day 2 after surgery). Two animals without clear evidence of MI were excluded from this study and humanely sacrificed by cervical dislocation after they were anesthetized with 3.5% trichloroacetaldehyde hydrate. Other twenty adult male BALB/c mice were succeeded in establishing myocardial infarction animal model. These animals were classified into four groups: Group 1 (n = 5; test group) mice received 1×10^7^–10^8^ pfu of Lenti-MLC-2v-VEGF_165_-IRES-NIS; Group 2 (n = 5; positive control group) mice received Lenti-EF1A- IRES-VEGF_165_-NIS; Group 3 (n = 5; negative control group): mice received 0.9% saline; Group 4 (n = 5 control group) mice received Lenti-MLC-2v-NIS, An outline of the experimental design is shown in Fig 1. On days 1, 3, 5, 7, 11, 21, 31, 45, 55 and 60, ^125^I SPECT/CT scanning was performed. We monitored the basic vital signs of each animal every day and measured their weight every 3 days. On day 60, we succeeded in all experimental procedure and then used humane endpoints to sacrifice animals according to our study design requirement. Animals were sacrificed humanely by cervical dislocation after they were anesthetized with 3.5% trichloroacetaldehyde hydrate. And then, we obtained animal heart samples to do immunohistochemistry analysis which were required by our study design.

### ^125^I and ^99m^Tc-MIBI imaging with micro-SPECT/CT in vivo

Micro-SPECT/CT scan system (Nano SPECT/CT) was used to image each mouse using the following conditions: scan speed, 60 s per frame; 18° per frame; slice thickness, 0.7–1 mm; pitch 0.5; CT scan, 55 kVp, 145 μA; voxel 146*146*146 μm; matrix 256*256; detector, 1–4 collimator pinhole; energy window, 35 ± 10% keV (^125^I), 140 ± 20%keV (^99m^Tc-MIBI). For each imaging session, mice were injected with 14.8–18.5 MBq of ^125^I (or ^99m^Tc-MIBI) via the tail vein after anesthetized and images were obtained by Micro-SPECT/CT software. Each mouse was anesthetized with 5% isofluorane in an induction chamber and then with 1–2% isofluorane gas delivered through a customized face-mask during each scanning.

As shown in Fig 1, on day one, ^125^I dynamic scanning was performed at 30 min, 60 min, 120 min and 24 h after the injection of ^125^I. The image quality was optimal at 60–120 min; on the following days (day 3, 5, 7, 11, 21, 31, 45, 55, 60), ^125^I SPECT/CT scanning was performed only at 60 min. On days 2 and 54, ^99m^Tc-MIBI SPECT/CT scanning was performed at 60 min according to the ^99m^Tc-MIBI scan routine.

Imaging was reconstructed by Invivoscope software after scanning, to achieve axial, sagittal and coronal SPECT, CT and fusion images. The radioactivity count (unit uCi/mm^3^) of ROI (regions of interest) was measured by InviCRO 3D software. The image interpretation of ^99m^Tc-MIBI and ^125^I SPECT/CT was based on the consensus of two nuclear medicine physicians and two PhD research fellows, who were blinded to any results of the other work-ups.

### Histology and immunohistochemistry

After animals were sacrificed humanely, we obtained animal heart samples to do immunohistochemistry analysis. We analyzed five samples from each group of group 1, 2 and 3. Explanted hearts (n = 15) were frozen, and embedded in methylcellulose. Serial short-axis cryosections (5-mm thick covering the entire heart at 1-mm intervals) were obtained for histological evaluation using a cryostat (Leica). The following primary antibodies and dilutions were used: anti-VEGF_165B_ antibody (1:50; Abcam ab90719), anti-NIS antibody (1:100; Abcam ab83816), and cell adhesion factor CD34 (1:500; Epitomics).

To estimate capillary density, three randomly selected fields near the peri-infarct region were evaluated. Computerized images were captured at a magnification of ×200. Capillary density (positive CD34 staining) was defined as the mean number of capillaries per square millimeter. Positive areas, Microvascular density (MVD) and integrated optical density (IOD value) were analyzed on Image ProPlus 6.0 software.

### Correlation between transgene expression and I-125 imaging

We calculated the mean I-125 uptake in ^125^I SPECT/CT imaging of each animal on day 1,3,7,21,45 and 60 (S1 Table). We also calculated the mean optical density (IOD value) of NIS (S2 Table), VEGF (S3 Table) and CD34 (S4 Table) in each transgene animal’s immunohistochemistry sample. Then we analyzed the correlation between mean I-125 uptake and mean IOD value of NIS, VEGF and CD34.

### Statistical analysis

All results were expressed as the mean ± SD. Statistical analysis was performed with SPSS13.0 software (SPSS Inc., IL, USA). Differences between multiple groups were compared with Paired-Samples Test with LSD multiple-comparison. The correlation between mean I-125 uptake and mean IOD value of NIS, VEGF and CD34 was analyzed with Pearson Bivariate Correlations. *P*-values less than 0.05 were considered statistically significant.

## Results

### Western blot analysis of dual-gene expression in H9C2 and C2C12 cells

**Fig 2:**
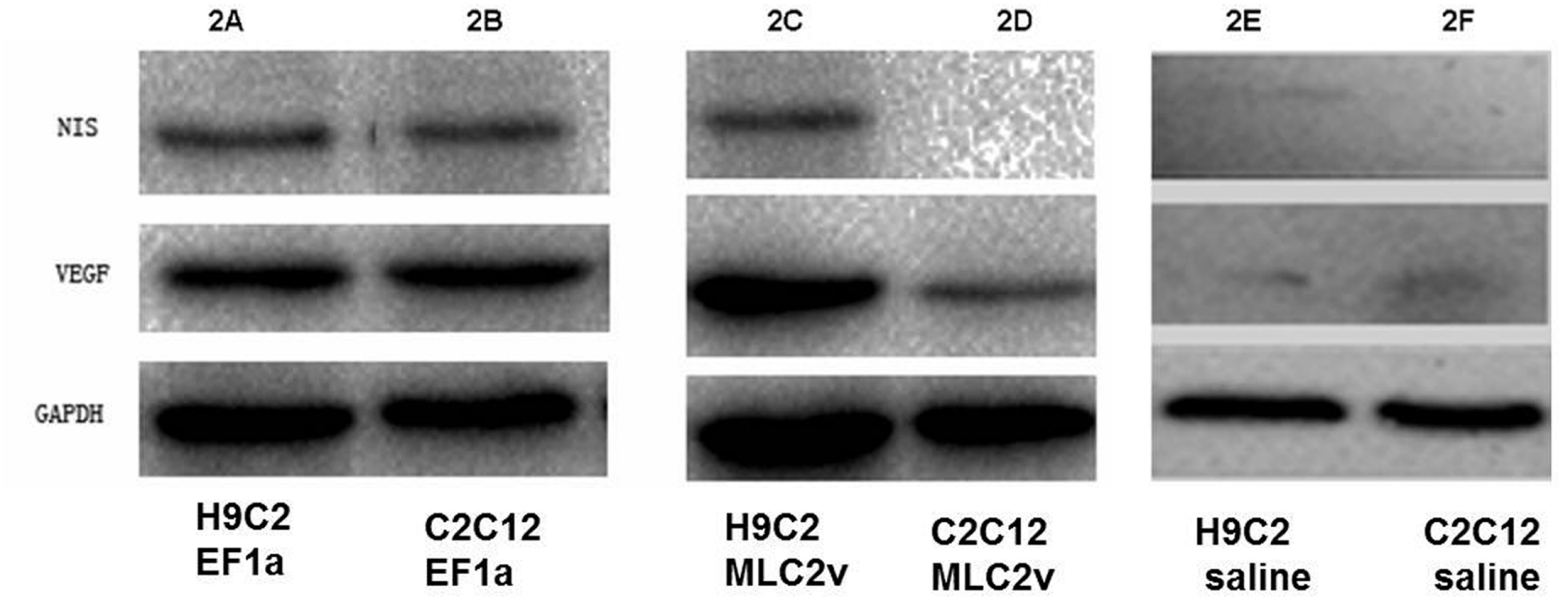
Western blot analysis of VEGF and NIS protein expression in H9C2 and C2C12 cells infected with lentivirus. (A, B) Both VEGF and NIS proteins were expressed at similar levels in H9C2 and C2C12 cells infected with Lenti-EF1a-VEGF_165_-IRES-NIS. (C, D) Following infection with Lenti-MLC-2v-VEGF_165_-IRES-NIS, proteins were also expressed in H9C2 cells; however, in C2C12 cells, NIS was not expressed and VEGF was expressed only at low levels. (E, F) In negative control group (saline), neither NIS nor VEGF was expressed.

Expression of the NIS and VEGF protein was analyzed by Western blot following the infection of H9C2 and C2C12 cells with Lenti-EF1a-VEGF_165_-IRES-NIS (Fig 2A and 2B), Lenti-MLC-2v-VEGF_165_-IRES-NIS (Fig 2C and 2D) and saline(Fig 2E and 2F). Both VEGF and NIS proteins were expressed at similar levels in H9C2 and C2C12 cells infected with Lenti-EF1a-VEGF_165_-IRES-NIS (Fig 2A and 2B). Following infection with Lenti-MLC-2v-VEGF_165_-IRES-NIS (Fig 2C and 2D), proteins were also expressed in H9C2 cells; however, in C2C12 cells, NIS was not expressed and VEGF was expressed only at low levels. In negative control group (saline), neither NIS nor VEGF was expressed (Fig 2E and 2F).

### ^99m^Tc-MIBI imaging with micro-SPECT/CT in vivo

**Fig 3:**
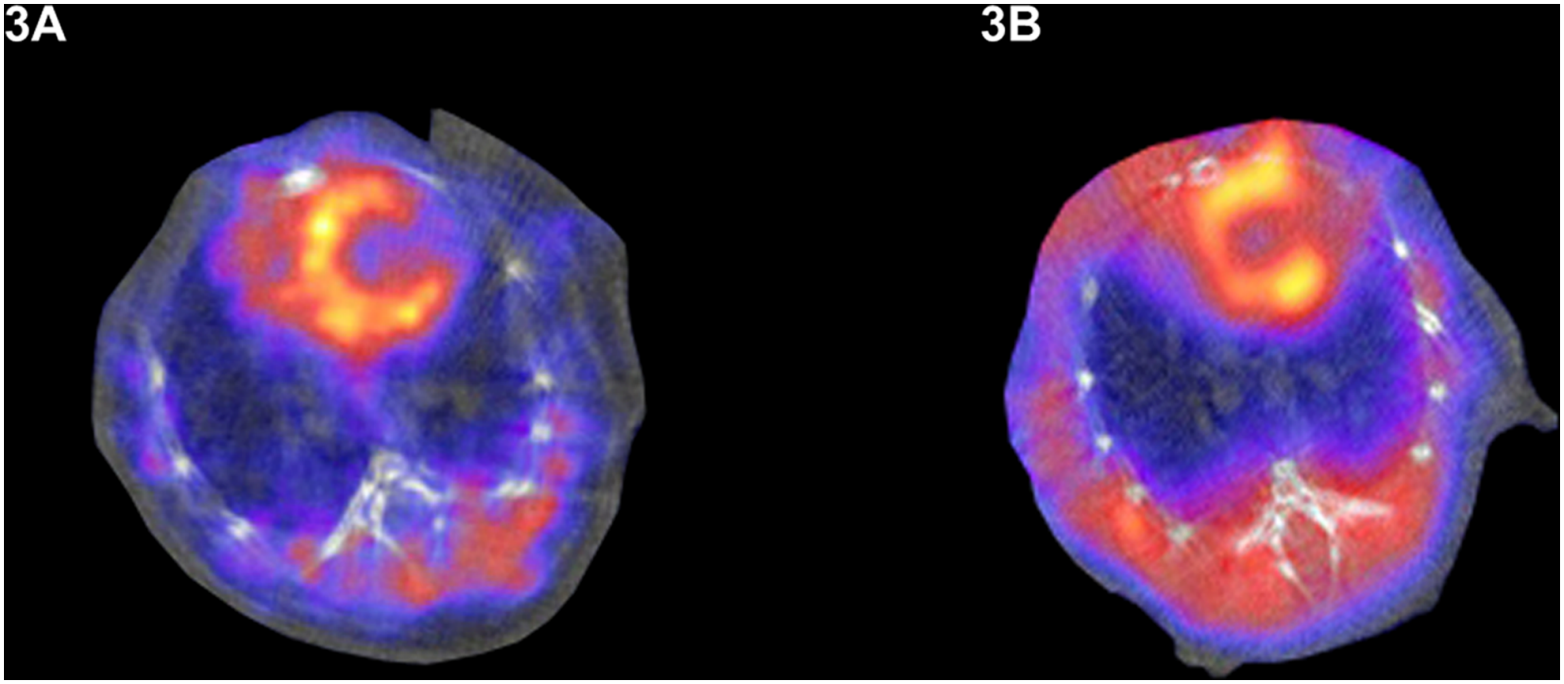
^99m^Tc-MIBI myocardial perfusion imaging. (A) ^99m^Tc-MIBI SPECT/CT scanning on day 2 after surgery to evaluate the extent of cardiac ischemia. Severely reduced uptakes in the apical, anterior and inferior cardiac wall were observed, which indicated that perfusion in these areas of the heart was successfully blocked. (B) On day 54, ^99m^Tc-MIBI SPECT/CT was repeated. In group 1 (test group, Lenti-MLC-2v-VEGF_165_-IRES-NIS), ^99m^Tc-MIBI SPECT/CT revealed improvements in perfusion and wall thickening in the apical anterior wall corresponding to the area of virus injection.

No procedural complications occurred in any mouse during any imaging procedure. All mice underwent ^99m^Tc-MIBI SPECT/CT scanning on day 2 after surgery to evaluate the extent of cardiac ischemia. Severely reduced levels of uptake in the apical, anterior and inferior wall were observed in 20 animals (Fig 3A), which indicated that perfusion in these areas of the heart had been successfully blocked. On day 54, ^99m^Tc-MIBI SPECT/CT was repeated. In group 1 (test group, Lenti-MLC-2v-VEGF_165_-IRES-NIS) (Fig 3B), ^99m^Tc-MIBI SPECT/CT revealed improvements in perfusion and wall thickening in the apical anterior cardiac wall, which corresponded to the area of virus injection. In groups 3 (negative control, 0.9% saline), ^99m^Tc-MIBI SPECT/CT revealed no improvements in the ischemia myocardium.

### Non-invasive monitoring of cardiac transgene expression in vivo

Myocardial ^125^I uptake was used for non-invasive monitoring of cardiac NIS expression in vivo on days 1, 3, 5, 7, 11, 21, 31, 45, 55, 60 following injection with recombinant lentivirus or saline (Fig 1).

**Fig 4:**
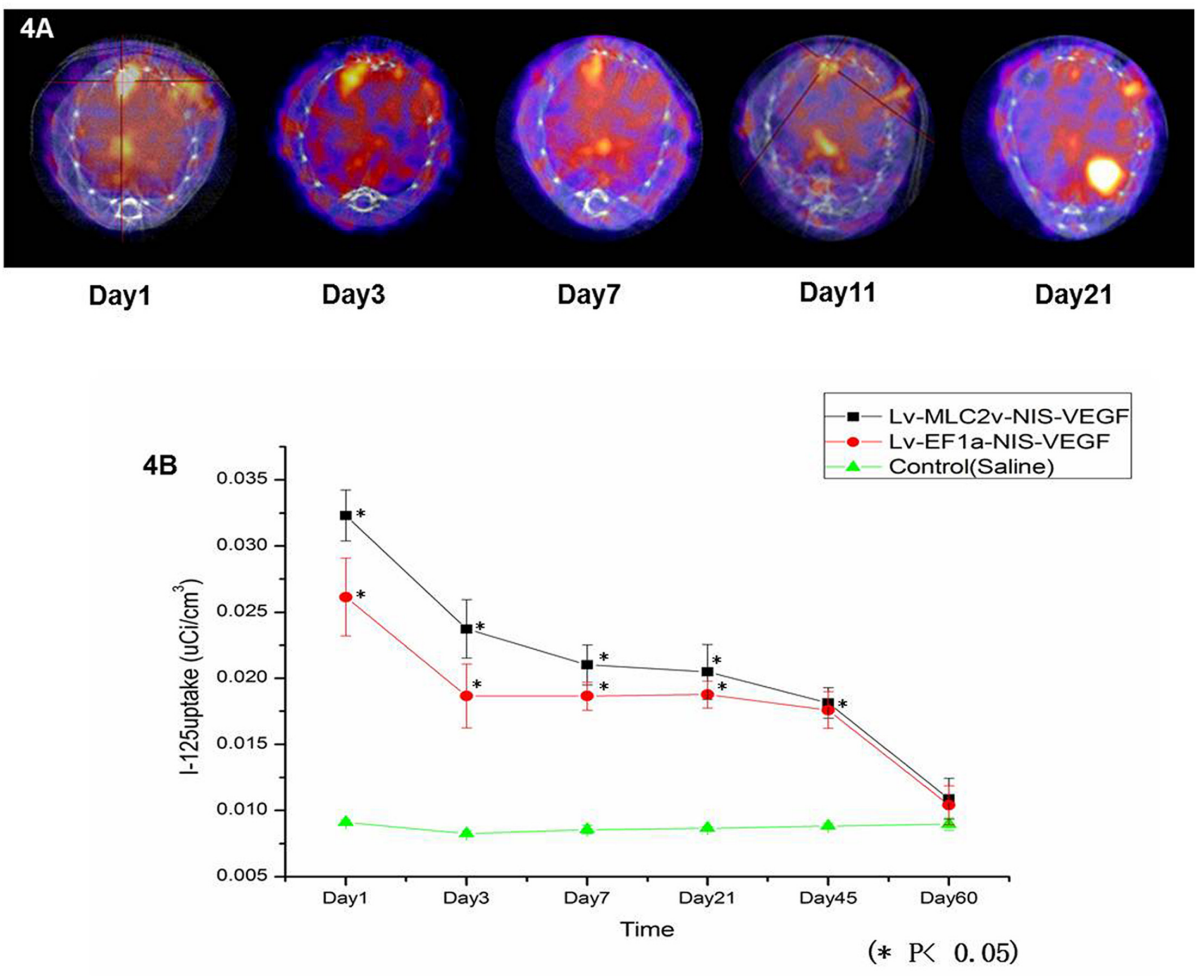
^125^I Imaging of cardiac transgene expression. (A) Axial cardiac ^125^I imaging of mice injected with Lenti-MLC-2v-VEGF_165_-IRES-NIS on days 1, 3, 7, 11 and 21. (B) Dynamic curve of cardiac ^125^I activity in the three groups. On day 1, mean ^125^I activity value in group 1 (0.0323±0.0019μCi/m^3^) was higher than that in group 2 (0.0261±0.0029μCi/m^3^) which had statically difference (*P* = 0.013<0.05). The mean ^125^I activity value in group 3 was (0.0091±0.0002) μCi/m^3^ which was statically lower than that in group 1 and 2 (P<0.01). On day 60, ^125^I uptakes in test and positive control groups became very low and no significant differences in the mean ^125^I activity values were detected between group 1 and group 2 (*P* = 0.531 > 0.05).

In group 1 (test group, n = 5), 1×10^7^–10^8^ pfu Lenti-MLC-2v-VEGF_165_-IRES-NIS were used for intramyocardial injection. Five mice underwent ^125^I SPECT/CT scanning on day one. ^125^I SPECT/CT imaging showed high cardiac ^125^I activity region (mean ^125^I activity value (0.0323±0.0019) μCi/m^3^) (Table 1) in the apical anterior wall which corresponded to the area of injection (Fig 4A). ^125^I activity value in group 1 was significantly higher than that in group 2 on day 1, 3 and 7 and higher than in group 3 on day 1, 3, 7,21 and 45 (*P*<0.05) (Table 2). On day 2, these mice underwent ^99m^Tc-MIBI SPECT/CT imaging. ^99m^Tc-MIBI imaging confirmed myocardial infarction lesion at apical anterior. Besides myocardium, other organs as thyroid, stomach and bladder also showed ^125^I physiology activity in ^125^I SPECT/CT scanning. Cardiac NIS transgene expression peaked at day 1 followed by a progressive decline to negligible levels on day 21 (Fig 4B). On day 21, mean ^125^I activity value was(0.0204±0.0020) μCi/m^3^ (Table 1) and became difficult to be recognized from SPECT/CT imaging.

**Table 1:** Mean±SD of I-125 Uptake in Different Groups.

Group	Group1	Lenti-mlc-VEGF-NIS	Group2	Lenti-EF1-VEGF-NIS	Group3	Saline
Time	Mean	±SD	Mean	±SD	Mean	±SD
**Day1**	**0.0323112**	**0.0019250**	**0.0261462**	**0.0029508**	**0.0091052**	**0.0002104**
**Day3**	**0.0237410**	**0.0021936**	**0.0186668**	**0.0024075**	**0.0082696**	**0.0002093**
**Day7**	**0.0210233**	**0.0015105**	**0.0186578**	**0.0010501**	**0.0085634**	**0.0003125**
**Day21**	**0.0204895**	**0.0020715**	**0.0187762**	**0.0010212**	**0.0086636**	**0.0001418**
**Day45**	**0.0181488**	**0.0011532**	**0.0176069**	**0.0013882**	**0.0088231**	**0.0002022**
**Day60**	**0.0108996**	**0.0015340**	**0.0104156**	**0.0014632**	**0.0089812**	**0.0004732**

Mean±SD of myocardial ^125^I uptake was measured on days 1, 3, 7, 21, 45, 60 following injection with recombinant lentivirus or saline in three groups mice (group 1 following injection with Lenti-MLC-2v-VEGF165-IRES-NIS, group 2 with Lenti-EF1A- IRES-VEGF165-NIS, Group 3 negative control group with saline)

**Table 2:** I-125 Uptake Differences between Three Groups (*P* value).

	Day1	Day3	Day7	Day21	Day45	Day60
Group1-Group2	0.015	0.008	0.013	0.167	0.56	0.531
Group1-Group3	0.00	0.00	0.00	0.00	0.00	0.079
Group2-Group3	0.00	0.001	0.00	0.00	0.00	0.095

^125^I activity value in group 1 was significantly higher than that in group 2 on day 1, 3 and 7 and higher than in group 3 on day 1, 3, 7,21 and 45 (*P*<0.05).

In group 2 (positive control group, n = 5), 1×10^7^–10^8^ pfu Lenti-MLC-2V-EF1a-VEGF_165_-IRES-NIS were used for injection. ^125^I SPECT/CT imaging showed high cardiac ^125^I activity region in the apical anterior wall corresponding to the area of injection. On day one, mean ^125^I activity value in group 2 was (0.0261±0.0029) μCi/m^3^ (Table 1). ^125^I activity value in group 2 was significantly lower than that in group 1(*P* = 0.013 < 0.05) (Fig 4B). On day 60, mean ^125^I activity values in group 1 and 2 became very low ((0.0108±0.0015) μCi/m^3^ in group 1 and (0.0104±0.0014) μCi/m^3^ in group 2) (Table 1) and no significant differences in the mean ^125^I activity values were detected between group 1 and group 2 (*P* = 0.531 > 0.05) (Table 2).

Group 3 (negative control group, n = 5) mice injected with saline. No specific accumulation of I-125 was observed in animal heart in ^125^I SPECT/CT scanning. Thyroid, stomach and bladder showed high ^125^I physiology activities. On day one, the mean ^125^I activity value in group 3 was (0.0091±0.0002) μCi/m^3^ which was statically lower than that in group 1 and 2 (Fig 4B, *P* < 0.01). On day 60, no significant differences in the mean ^125^I activity values were detected among three groups (*P* > 0.05) (Table 2).

### Histology and immunohistochemistry

**Fig 5:**
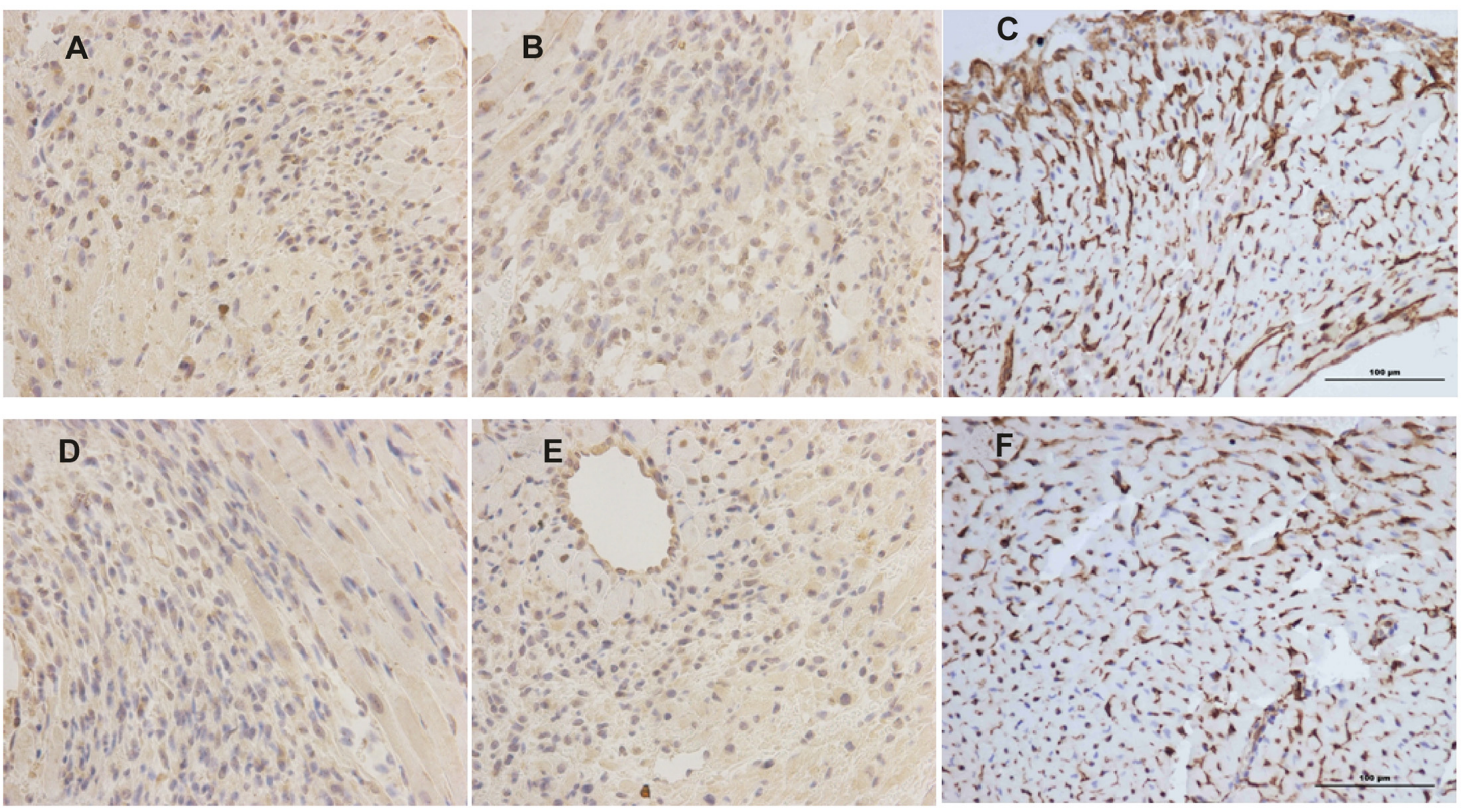
Immunohistochemical analysis of NIS, VEGF and CD34. (A-C) Myocardial expression of NIS, VEGF and CD34 following infection with Lenti-MLC-2v-VEGF_165_-IRES-NIS were confirmed by immunohistochemistry which is shown as brown-yellow spots (magnification, x400). (D-F) Low levels expression of NIS, VEGF, and CD34 was detected in the negative control group.

**Fig 6:**
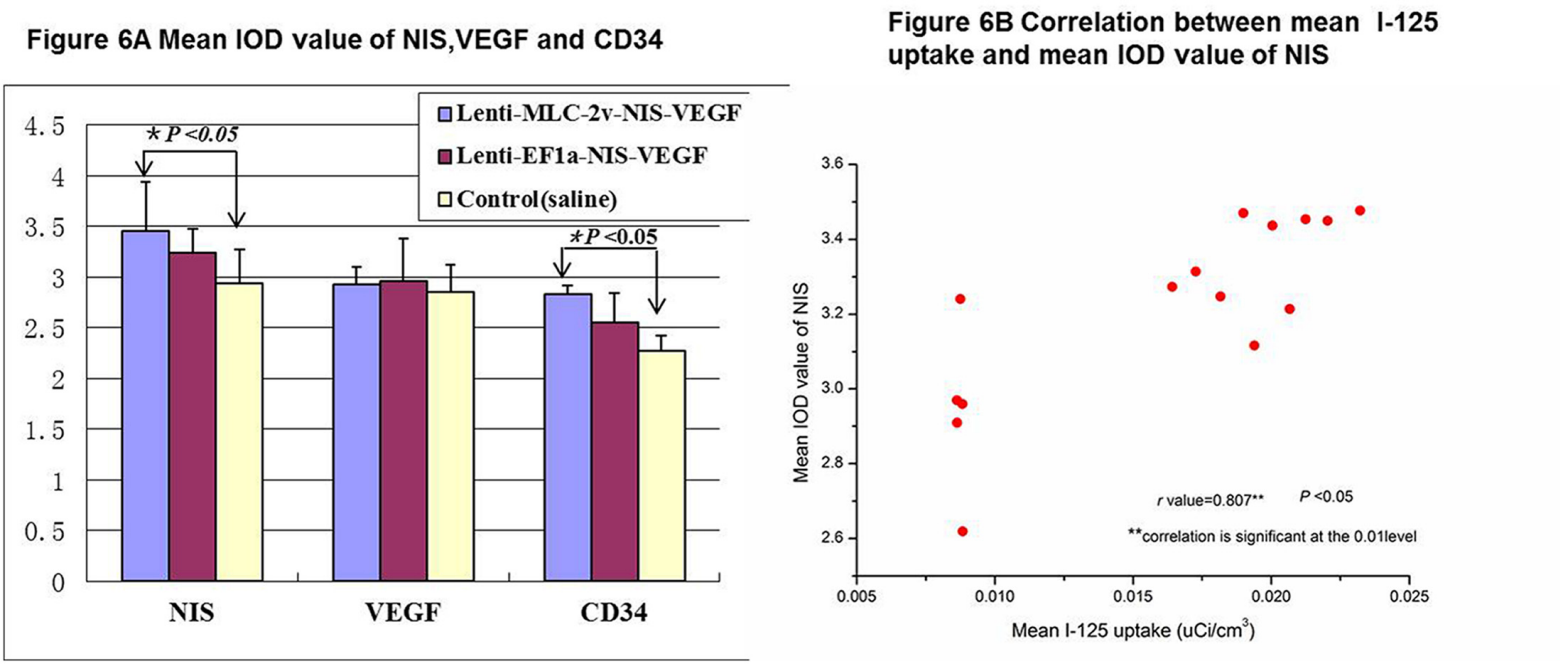
Correlation between transgene expression and I-125 imaging. (A) Mean IOD values of NIS, VEGF and CD34. No significant differences in the mean IOD values of NIS, VEGF and CD34 were detected between group 1 (MLC-2v promoter) and group 2 (EF1a promoter) (*P* > 0.05). Expression of NIS and CD34 in group 1 (MLC-2v promoter) was significantly higher than that in the control group (*P* < 0.05). (B) Correlation between mean I-125 uptake and mean IOD value of NIS. Mean I-125 uptake was significantly correlated with mean IOD value of NIS (Pearson Correlation value *r* = 0.807**, *P*<0.05).

The cardiac infarct areas shown by ^99m^Tc-MIBI SPECT/CT imaging in vivo were confirmed by ex vivo hematoxylin and eosin staining. Myocardial expression of NIS, VEGF and CD34 following infection with Lenti-MLC-2v-VEGF_165_-IRES-NIS can be detected by immunohistochemistry which is shown as brown-yellow spots (Fig 5A–5C). Low levels expression of NIS, VEGF, and CD34 was detected in the negative control group (Fig 5D–5F) with mean integrated optical density (IOD) values of 2.9400 ± 0.3301, 2.8550 ± 0.2711 and 2.2767 ± 0.1457. No significant differences in the mean IOD values of NIS, VEGF and CD34 were detected between group 1 (MLC-2v promoter) and group 2 (EF1a promoter) (*P* > 0.05), mean IOD values of NIS, VEGF and CD34 in group 1 were 3.4570±0.4808, 2.9276±0.1681, 2.8267±0.0945 and in group 2 were 3.2345±0.2428, 2.9570±0.4211, 2.5467±0.2936. Expression of NIS and CD34 in group 1 (MLC-2v promoter) was significantly higher than that in the control group (*P* < 0.05, Fig 6A).

### Correlation between transgene expression levels and imaging findings

Mean I-125 uptake was significantly correlated with mean IOD value of NIS (Pearson Correlation value *r* = 0.807**, *P* <0.05, Fig 6B) and also significantly correlated with mean IOD of CD34 (Pearson Correlation value *r* = 0.807**, *P* <0.05). Mean I-125 uptake showed no significant correlation with mean IOD value of VEGF (Pearson Correlation value *r* = 0.370, *P* >0.05) (Table 3).

**Table 3:** Correlation between I-125 uptake and Mean IOD value of NIS, VEGF,CD34.

		Mean IOD value of NIS	Mean IOD value of VEGF	Mean IOD value of CD34
**I-125 uptake**	**Pearson correlation value**	0.807**	0.370	0.884**
**(uCi/cm^3^)**	***P* value (Sig.)**	0.000	0.175	0.000

**Table 3** showed mean I-125 uptake was significantly correlated with mean IOD value of NIS (Pearson Correlation value *r* = 0.807**, *P* <0.05) and also significantly correlated with mean IOD of CD34 (Pearson Correlation value *r* = 0.807**, *P* <0.05).

(** Correlation is significant at the 0.01 level (2-tailed).)

## Discussion

In this study, we demonstrated the feasibility of noninvasive SPECT/CT imaging using NIS as the reporter gene to monitor (up to 60 days) the expression of the therapeutic gene VEGF, in a mouse model of myocardial ischemia. Cardiac NIS transgene expression peaked on day 1 followed by progressive decline to baseline levels at day 21. The test group injected with Lenti-MLC-2v-VEGF_165_-IRES-NIS showed the highest levels of ^125^I activity and the positive group injected with Lenti-EF1a-VEGF_165_-IRES-NIS showed the lower ^125^I activity levels. No specific accumulation of I-125 was observed in animal heart in negative control group.

Non-invasive methods to monitor gene expression and location are crucial for the success of gene therapy. HSV1-tk and the sodium/iodide symporter (NIS) are promising radionuclide imaging reporter genes. Recently, Perin et al. [9] used HSV1-tk for the long-term (up to 5 months) in vivo monitoring of stem cells. Compared with HSV1-tk, NIS does not require complex radiochemistry labels and can be used with imaging techniques such as SPECT. This facilitates its use in hospitals that lack radiochemistry laboratories and PET facilities.

To date, some reports have demonstrated the feasibility of using NIS for myocardial gene expression imaging in animals using scintigraphy or SPECT/CT [20–24], but these studies did not used a combination of NIS with the therapeutic gene. Our study is the first to use a dual gene recombinant lentivirus, which linked NIS to VEGF. We identified NIS and VEGF co-expression both in vitro and vivo. Our study also confirmed the correlation between transgene expression levels and imaging findings. We proved mean I-125 uptake was significantly correlated with mean IOD value of NIS and CD34 (Table 3). Unfortunately, we failed to find significant correlation between I-125 uptake and mean IOD value of VEGF. We think the reason may be the complexity of VEGF molecular mechanism in vivo and we already confirmed NIS and VEGF co-expression in vitro. In future work, we hope to make progress by increasing animal number and using new gene introducing technique. The significant correlation between I-125 uptake and mean IOD value of NIS proved ^125^I SPECT/CT imaging was sensitive to monitor NIS gene expression in vivo. CD34 antibody is one of the most studied vascular markers which is important for the prognostic evaluation of patients and also has diagnostic value to reflect neoangiogenesis activity. The correlation between I-125 uptake and mean IOD value of CD34 showed ^125^I SPECT/CT imaging was helpful to monitor improvements in myocardial neoangiogenesis. These results were encouraging for further investigations of the application of NIS to monitor the magnitude, location and duration of VEGF expression, and those of other potential therapeutic proteins, such as FGF, HGF and HIF-1.

The success of gene therapy depends on gene transfer vectors that facilitate the expression of a therapeutic gene. Viral vectors are efficient tools for genetic modification in vitro and in vivo. In this study, we chose a lentivirus system for the gene transfer vector in order to obtain longer term NIS expression. Lee et al. [24] used a dual-gene adenovirus (Ad-EGFP-NIS) to assess myocardial gene expression in living mice and ^123^I imaging demonstrated clear focal myocardial uptake at the injection site between days 2 and 4 that was no longer visible by day 9. In our study, we used ^125^I micro-SPECT/CT reporter imaging which showed higher levels of uptake in the mouse myocardium where NIS expression peaked on day 1 and was still visible on day 21. The prolonged expression of NIS is very helpful for gene therapy studies because the therapeutic effect cannot be observed within a short time period.

Specificity is an important concept for cardiac gene therapy. A specific promoter can restrict the expression of NIS only in target cells and reduce unnecessary radioiodine uptake in normal tissues; this increases specific cardiac transgene expression and therapeutic function [25]. Su et al. [26] evaluated the use of the cardiac MLC-2v promoter and the hypoxia response element to mediate cardiac-specific and hypoxia-inducible VEGF expression. We chose the first 250bp of the MLC-2v promoter because this contractile protein is abundant in cardiac muscles [17]. Although the MLC-2v promoter is 3 kb long, critical elements that mediate cardiac-specific gene expression are located within the first 250bp [18]. In our study, we showed high levels of NIS and VEGF protein expression following the in vitro infection of H9C2 cardiomyocytes with recombinant lentiviruse that encoded the genes expressed from the cardiac-MLC-2v promoter, while expression levels in non-cardiac C2C12 cells were low. The in vivo imaging of mice injected with Lenti-MLC-2v-VEGF_165_-IRES-NIS showed higher levels of cardiac ^125^I activity than mice injected with Lenti-EF1a-VEGF_165_-IRES-NIS. On day 1, mean ^125^I activity value in group 1 was higher than that in group 2 (*P* < 0.05). The mean ^125^I activity value in group 3 was statically lower than that in group 1 and 2 (*P* < 0.01). On day 60, ^125^I uptakes in test and positive control groups became very low and no significant differences in the mean ^125^I activity values were detected between group 1 and group 2 (*P* = 0.531 > 0.05).

On day 2, severely reduced levels of uptake in the apical, anterior and inferior wall were observed in ^99m^Tc-MIBI SPECT/CT imaging in animal cardiac. On day 54, ^99m^Tc-MIBI SPECT/CT was repeated. In group 1 (test group, Lenti-MLC-2v-VEGF_165_-IRES-NIS) (Fig 3B), ^99m^Tc-MIBI SPECT/CT revealed improvements in the perfusion and wall thickening in cardiac wall, which corresponded to the area of virus injection and no cardiac improvements were observed in group 3. Myocardial expressions of NIS, VEGF following infection with Lenti-MLC-2v-VEGF_165_-IRES-NIS were confirmed by immunohistochemistry. According to these results, we confirmed our hypothesis recombinant lentivirus Lenti-MLC-2v-VEGF_165_-IRES-NIS can non-invasive monitor gene therapy effectiveness in the treatment of cardiac ischemia in vivo by SPECT imaging.

Our study had certain limitations. We hypothesized that simple gene viral therapy would limit the therapeutic and imaging efficacy. Simple gene therapy can induce immune responses and always has poor rates of treatment efficacy [12]. Combined therapeutic gene transfection with stem cell therapy may improve the treatment effect and also prolong gene expression durations [9]. We expect our future work will combine gene transfection with stem cell therapy to prolong gene expression durations and give more satisfactory results.

## Conclusions

We have successfully demonstrated the co-expression of NIS and VEGF from the same recombined lentiviral vector plasma, as well as the feasibility of monitoring the magnitude, location and duration of therapeutic gene expression indirectly by imaging NIS reporter gene expression. Based on this study, we hope to develop an effective method for monitoring gene therapy in experimental or clinical studies of myocardial ischemic disease.

## Supporting Information

S1 File Copy of the 3R ARRIVE guidelines for reporting animal research. Our study was performed according to requirement of the ARRIVE guidelines for reporting animal research. We uploaded a copy of the completed ARRIVE checklist as supplemental information in S1 File.
(PDF)

S1 Table Dataset of I-125 uptake in different groups. S1 Table was the dataset of ^125^I uptake which was measured on days 1, 3, 7, 21, 45, 60 following injection with recombinant lentivirus or saline in three groups mice (group 1 following injection with Lenti-MLC-2v-VEGF165-IRES-NIS, group 2 with Lenti-EF1A- IRES-VEGF165-NIS, Group 3 negative control group with saline).
(XLS)

S2 Table Dataset of NIS mean IOD value. (XLS)

S3 Table Dataset of VEGF mean IOD value. (XLS)

S4 Table Dataset of CD34 mean IOD value. (XLS)
